# Metabolic Changes Associated With Muscle Expression of SOD1^G93A^

**DOI:** 10.3389/fphys.2018.00831

**Published:** 2018-07-10

**Authors:** Gabriella Dobrowolny, Elisa Lepore, Martina Martini, Laura Barberi, Abigail Nunn, Bianca Maria Scicchitano, Antonio Musarò

**Affiliations:** ^1^Laboratory affiliated to Istituto Pasteur – Fondazione Cenci Bolognetti, DAHFMO – Unit of Histology and Medical Embryology, Sapienza University of Rome, Rome, Italy; ^2^Center for Life Nano Science, Istituto Italiano di Tecnologia, Rome, Italy; ^3^Istituto di Istologia ed Embriologia, Università Cattolica del Sacro Cuore, Fondazione Policlinico Universitario Agostino Gemelli, Rome, Italy

**Keywords:** skeletal muscle, SOD1^G93A^, muscle fiber types, metabolic alterations, ALS, oxidative stress

## Abstract

Amyotrophic lateral sclerosis (ALS) is a severe neurodegenerative disorder, classified into sporadic or familial forms and characterized by motor neurons death, muscle atrophy, weakness, and paralysis. Among the familial cases of ALS, approximately 20% are caused by dominant mutations in the gene coding for superoxide dismutase (SOD1) protein. Of note, mutant SOD1 toxicity is not necessarily limited to the central nervous system. ALS is indeed a multi-systemic and multifactorial disease that affects whole body physiology and induces severe metabolic changes in several tissues, including skeletal muscle. Nevertheless, whether alterations in the plasticity, heterogeneity, and metabolism of muscle fibers are the result of motor neuron degeneration or alternatively occur independently of it remain to be elucidated. To address this issue, we made use of a mouse model (MLC/SOD1^G93A^) that overexpresses the SOD1 mutant gene selectively in skeletal muscle. We found an alteration in the metabolic properties of skeletal muscle characterized by alteration in fiber type composition and metabolism. Indeed, we observed an alteration of muscle glucose metabolism associated with the induction of Phosphofructokinases and Pyruvate dehydrogenase kinase 4 expression. The upregulation of Pyruvate dehydrogenase kinase 4 led to the inhibition of Pyruvate conversion into Acetyl-CoA. Moreover, we demonstrated that the MLC/SOD1^G93A^ transgene was associated with an increase of lipid catabolism and with the inhibition of fat deposition inside muscle fibers. All together these data demonstrate that muscle expression of the SOD1^G93A^ gene induces metabolic changes, along with a preferential use of lipid energy fuel by muscle fibers. We provided evidences that muscle metabolic alterations occurred before disease symptoms and independently of motor neuron degeneration, indicating that skeletal muscle is likely an important therapeutic target in ALS.

## Introduction

Amyotrophic Lateral Sclerosis (ALS) is a multi-factorial and multi-systemic disease due to severe alterations in several tissues and cell compartments, such as motor neurons, glia, and muscle (Wijesekera and Leigh, 2009). In addition, energy balance is severely compromised in ALS patients, owing to higher consumption than intake with increased resting energy use, along with abnormal lipid metabolism (Kasarskis et al., 1996; Desport et al., 2005; Dupuis et al., 2008; Bouteloup et al., 2009; Funalot et al., 2009).

Interestingly, while lower body fat represents an ALS premorbidity factor (Gallo et al., 2013), overweight or obese people have a lower risk to develop ALS (O’Reilly et al., 2013). A recent study has pointed out that ALS patients lose weight about 10 years before motor symptoms (Peter et al., 2017) and have higher daily energy intake to compensate the higher energy consumption that has been documented before clinical onset of the disease (Huisman et al., 2015).

Similar energetic alterations were found in transgenic animal models of ALS that ubiquitously express the mutant isoform of the gene coding for superoxide dismutase 1 (SOD1^G93A^) (Gurney et al., 1994). Indeed, the SOD1^G93A^ mice are characterized by increased energy expenditure and by a concomitant skeletal muscle hypermetabolism (Dupuis et al., 2004). It has been demonstrated that a diet rich in lipids can delay disease onset and motor neuron degeneration and can extend life expectancy of mutant SOD1 mice (Dupuis et al., 2004).

During disease progression and muscle denervation, the SOD1^G93A^ mice exhibit a loss of fast-twitch glycolytic fibers and show a transition of skeletal myosin from fast glycolytic type toward slower oxidative one (Peggion et al., 2017). The loss of fast glycolytic fibers is considered a consequence of the degeneration of fast fatigable motor neuron synapsis that leads to a shift in muscle fiber type. In addition, skeletal muscle of ALS patients and transgenic ALS mouse models show mitochondrial functional impairment (Carri and Cozzolino, 2011); this mitochondria alteration together with increased energy expenditure might represent one of the first targets of ALS pathology, retrogradely affecting the nerve in a sort of dying back phenomenon (Shi et al., 2010).

It has been recently demonstrated, in the muscles of SOD1^G93A^ mouse model, that the metabolic switch toward lipid use represents an early pathological event, suggesting that the metabolic defect is distinct from denervation (Palamiuc et al., 2015). However, the animal model chosen to conduct the study did not help to definitely disclose whether muscle metabolic changes and neuromuscular junction alterations, two pathogenic events associated with ALS, are a consequence of changes in synapsis functionality or are independent of it.

In previous works we have demonstrated that muscle-selective expression of SOD1 mutation (MLC/SOD1^G93A^), induces alterations in the contractile apparatus, and causes mitochondrial dysfunction without affecting motor neuron survival (Dobrowolny et al., 2008). Recently, we found a significant reduced stability of muscles mitochondrial membrane potential, and we observed a reduced integrity of mitochondrial network in the region of the Neuromuscular Junctions (NMJ) of MLC/SOD1^G93A^ transgenic mice, suggesting that mitochondrial alterations and accumulation of oxidative stress negatively impact on NMJ stability (Dobrowolny et al., 2018).

Here, taking advantage of MLC/SOD1^G93A^ mice, we investigated whether muscle specific accumulation of SOD1^G93A^ can induce metabolic changes that occur independently from motor neuron degeneration and precede muscle denervation.

## Materials and Methods

### Mice

Four-month-old MLC/SOD1^G93A^ mice overexpressing the mutant SOD1 gene (SOD1^G93A^) under the control of the Myosin Light Chain (MLC) muscle specific promoter (Dobrowolny et al., 2008) and 4-month-old Friend leukemia virus B (FVB) (Jackson Laboratories) have been used. Male and female mice were used indiscriminately. The animals were housed in a temperature controlled (22°C) room with a 12:12 h light–dark cycle and housed in a number of three to five per cage. All animal experiments were approved by the ethics committee of Sapienza University of Rome-Unit of Histology and Medical Embryology and were performed in accordance with the current version of the Italian Law on the Protection of Animals.

### Histological Analysis

Segments of tibialis anterior (TA) muscles isolated from both wild type (Wt) and MLC/SOD1^G93A^ transgenic mice were embedded in tissue freezing medium and snap frozen in nitrogen-cooled isopentane. Ten μm sections were prepared for either NADH-transferase or PAS staining. Images were collected using a Zeiss AX10-Imager A2 connected to the Axiocam 503 color.

### RNA Preparation and Real-Time Analysis

Total RNA from Wt and MLC/SOD1^G93A^ transgenic muscles was isolated from tibialis anterior muscles (TA) by TRIzolTM reagent (Thermo Fisher Scientific). The yield, quality, and integrity of RNA were determined using NanoDrop ND-2000 (Thermo Fisher Scientific).

Total RNA (1 μg) reverse-transcription was performed using Qiagen Reverse Trascription Kit (Qiagen) whereas 10 ng of RNA was reverse transcribed using the TaqMan micro-RNA Reverse Transcription Kit (Thermo Fisher Scientific). Quantitative PCR was performed using the ABI PRISM 7500 SDS (Thermo Fisher Scientific), TaqMan universal MMIX II (Thermo Fisher Scientific), and TaqMan probe (Thermo Fisher Scientific). Quantitative RT-PCR sample value was normalized for the expression of β-actin and U6 snRNA for mRNA and microRNA, respectively. The relative expression was calculated using the 2^−ΔΔ*C*_t_^ method (Livak and Schmittgen, 2001) and reported as fold change.

### Protein Extraction and Western Blot Analysis

Protein extraction from both Wt and MLC/SOD1^G93A^ transgenic tibialis anterior muscles was performed in Sodium Chloride, 1 mM Phenylmethylsulfonyl fluoride, 1 μg/ml Aprotinin, 1 μg/ml Leupeptin, 1 μg/ml Pepstatin, 1 mM Sodium orthovanadate, 1 mM Sodium fluoride. Equal amounts of protein from each muscle lysate were separated in SDS polyacrylamide gel and transferred onto a nitrocellulose membrane. Filters were saturated with 5% milk and then blotted with antibodies against Slow myosin (1:3000) (Sigma Aldrich, United States), Glut4 (1:250) (Cell Signaling Technology, United States), PDH-E1α (pSer300) (1:400) (Calbiochem, United States and Canada), Pyruvate Dehydrogenase complex (PDH; 1:600) (Cell Signaling, United States), Phospho-GSk3β (pSer9) and total GSk-3β (1:1000) (Cell Signaling, United States), ATGL (1:100) (Cell Signaling, United States), pACC(1:600) (Cell Signaling, United States), ACC(1:250) (Cell Signaling, United States), Plin2 (2μg/ml) (Life Span Biosciences, United States), and α-tubulin (1:2000) (Sigma Aldrich, United States).

Then, filter was incubated with secondary antibodies Goat anti-mouse IgG HRP-conjugated (1:7000) (Bethyl, Montgomery, TX, United States) or Goat anti-rabbit IgG HRP-conjugated (1:7000) (Bethyl, Montgomery, TX, United States) in 1% milk for 1 h. All the antibodies were chosen as validated for western blot by manufactures.

### Glucose Tolerance Test

Four-month-old Wt and MLC/SOD1^G93A^ transgenic mice were fasted for 18 h and blood was drawn from a small incision at the tip of the tail. Blood glucose levels were evaluated using a commercial Glucose multicare (BSI) kit. Glucose tolerance test (GTT) was performed measuring glucose before and after IP injection of 2 g/kg body mass of glucose. Changes in blood glucose were followed for 120 min with measurements taken every 30 min.

### CARS Microscopy

A multi modal non-linear microscope was used to record 3D stacks of images in cryosections of muscle tissue, using the strong CH vibration at 2840 cm^−1^ of lipids as image contrast. In brief, a picosecond laser source (Levante Emerald OPO, APE Angewandte Physik & Elektronik GmbH, Germany, pumped by an Nd:Vanadate laser at 532 nm, High Q Laser GmbH, Austria) generated two pulses at 76 MHz repetition rate, with powers of 50 mW and 120 mW for the pump (817 nm) and Stokes (1064 nm) beams, respectively, that were spatially and temporally overlapped and then coupled to a modified inverted laser scanning confocal microscope (Nunn et al., 2016).

### Statistical Analysis

All details related to statistical tests, statistical parameters, including sample sizes (*n* = number of animal subjects per group), and significance are reported in Figure Legends.

For real-time PCR we considered sample size adequate when the two groups were significantly different (*P* < 0.05). Unless otherwise indicated, *P*-values for simple pair-wise comparisons were performed using a two-tailed unpaired and non-parametric Mann–Whitney test and graph values are reported as mean ± SEM. (error bars).

Data is considered statistically significant when *p* < 0.05. Asterisks in figures, indicate statistical significance ^∗^*p* < 0.05, ^∗∗^*p* < 0.05. All statistical analysis was performed using GraphPad PRISM 6 software.

## Results

### MLC/SOD1^G93A^ Mice Exhibit a Fast-to-Slow Shift in the Fiber Type Composition

**FIGURE 1:**
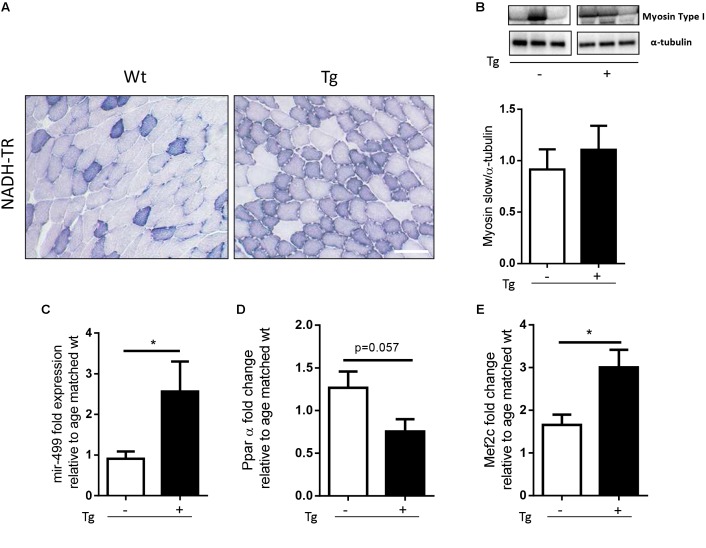
MLC/SOD1^G93A^ mice exhibit a fast-to-slow switch in muscle fiber composition. **(A)** NADH-TR staining in Wt and Tg TA muscles shows a shift from glycolitic to oxidative metabolism (darker fibers), Bar, 100 μm. **(B)** Representative western blot analysis of Myosin type I protein expression in both Wt and Tg mice. Lower panel shows densitometric analysis for Myosin type I expression relative to α-tubulin in Wt and Tg mice (Wt, Tg *n* = 5,4). **(C–E)** Real-time PCR analysis of miR-499 **(C)**, PPARα **(D)**, Mef2c **(E)** transcript in Wt and Tg mice [*p* = 0.0177 Wt, Tg *n* = 7,5 **(C)**; *p* = 0.0571 Wt, Tg *n* = 3,4 **(D)**; ^∗^*p* = 0.0266 Wt, Tg *n* = 8,10 **(E)**]. Data are represented as mean ± SEM. In **(B)** the lanes were run on the same gel but were non-contiguous.

In a previous work we have demonstrated that localized expression of SOD1^G93A^ promoted the fiber-type switching from glycolytic toward more oxidative fibers in EDL muscle of MLC/SOD1^G93A^ mice (St. Pierre et al., 2006). To further support this evidence, we analyzed NADH levels in the tibialis anterior (TA) muscle, which contains high proportion of the fastest glycolytic fibers. **Figure 1A** shows higher content of NADH in the TA muscle of MLC/SOD1^G93A^ mice compared to that observed in the TA of Wt littermates.

To further investigate the metabolic profile of the MLC/SOD1^G93A^ muscles we assayed the expression of Myosin Slow type I levels, and we observed a slight increase of Myosin Slow type I in the transgenic muscles compared to the Wt ones (**Figure 1B** and Supplementary Figure S1).

Recent evidences have demonstrated that the nuclear receptors PPARβ/δ and PPARα play opposing roles upon the type I fiber specification program, regulating the expression of two non-coding RNA, MiR-208b, and MiR-499, both activating a signaling cascade for the expression of muscle slow twitch contractile proteins (van Rooij et al., 2009). In particular, it has been demonstrated that genetic ablation of PPARα or the up-regulation of PPARβ/δ mediated by PGC-1α, lead to significant accumulation of MiR-208b and MiR-499 and specify for a slow muscle fiber program (Gan et al., 2013).

We analyzed the expression level of PPARα and MiR-499 in both Wt and MLC/SOD1^G93A^ (Tg) TA muscles and we observed a significant down-modulation of PPARα expression levels and a concomitant up-regulation of MiR-499 (**Figures 1C,D**) in MLC/SOD1^G93A^ mice compared to Wt littermates, suggesting that muscle specific expression of SOD1 mutant gene triggers a slow-oxidative program mediated by a PGC1α/PPAR dependent regulatory circuit.

A key regulator of PGC-1α expression is Mef2c, a transcriptional factor that is responsible for fast-to-slow switch of muscle fibers (Czubryt et al., 2003). We revealed a significant increased expression of Mef2C transcript in the muscle of MLC/SOD1^G93A^ mice compared to that of the Wt littermates (**Figure 1E**), confirming the evidences of a metabolic transition of muscle fibers from glycolytic toward more oxidative type.

Overall these data indicate that perturbation in redox signaling cascades, induced by muscle specific expression of SOD1^G93A^, promoted a muscle metabolic adaptation in line with what observed in the skeletal muscle of SOD1^G93A^ mice that ubiquitously express the mutant SOD1 gene (Palamiuc et al., 2015).

### Muscle Glucose Metabolism Is Altered in the MLC/SOD1^G93A^ Mice

**FIGURE 2:**
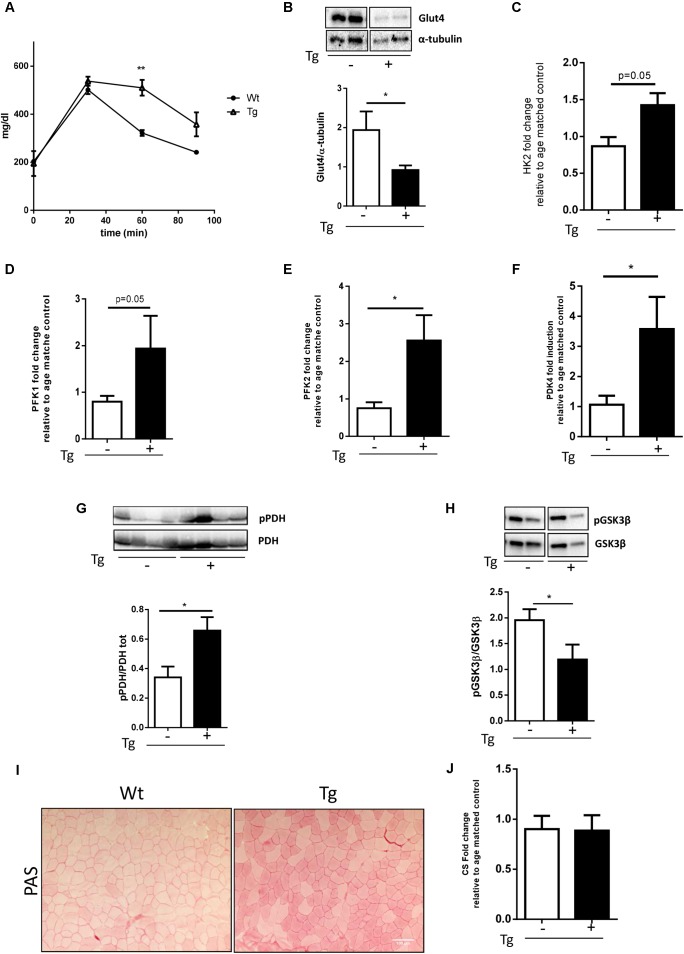
Muscle glucose metabolism is altered in MLC/SOD1^G93A^ mice. **(A)** Analysis of blood glucose levels in Wt and Tg mice for 120 min (^∗∗^*p* < 0.01 Wt, Tg *n* = 3,3; Two-way ANOVA test Time *p* < 0.0001, Interaction *p* = 0.0003). **(B)** Representative western blot analysis of Glut4 protein expression in both Wt and Tg mice. Lower panel shows densitometric analysis for Glut4 expression relative to α-tubulin in Wt and Tg mice (*t*-test ^∗^*p* = 0.0285 Wt, Tg *n* = 9,12); **(C–F)** Real-time PCR analysis of HK2 **(C)**, PFK1 **(D)**, PFK2 **(E)**, PDK4 **(F)** transcripts in Wt and Tg mice [*t*-test *p* = 0.0532 Wt, Tg *n* = 3,3 **(C)**; *p* = 0.0571 Wt, Tg *n* = 4,3 **(D)**; ^∗^*p* = 0.0244 Wt, Tg *n* = 9,9 **(E)**; ^∗^*p* = 0.0482 Wt, Tg *n* = 9,11 **(F)**]; **(G)** Representative western blot analysis of pPDH/PDH protein expression in both Wt and Tg mice. Lower panel shows densitometric analysis for pPDH expression relative to PDH in Wt and Tg mice (*t*-test ^∗^*p* = 0.0261 Wt, Tg *n* = 5,5); **(H)** Representative Western blot analysis of PGSKβ and GSKβ protein expression in both Wt and Tg mice. Lower panel shows densitometric analysis for PGSKβ expression relative to total GSKβ content normalized for protein loading in Wt and Tg mice (*t*-test ^∗^*p* = 0.0476 Wt, Tg *n* = 10,10; **(I)** representative microphotographs of PAS staining from Wt and Tg TA muscles. Bar, 100 μm. **(J)** Real-time PCR analysis of CS (Wt, Tg *n* = 4,4). Data are represented as mean ± SEM. In **(B,G,H)** the lanes were run on the same gel but were noncontiguous.

Glycolysis is a metabolic pathway that converts glucose into pyruvate, providing high energy substrate. Glycolysis is dysregulated in the animal model of ALS, the SOD1^G93A^ mice that ubiquitously overexpress the mutant form of the SOD1 gene (Palamiuc et al., 2015). To verify whether glucose metabolism was also altered in the MLC/SOD1^G93A^ mice, we performed the glucose tolerance test, demonstrating that transgenic mice, compared with Wt littermates, presented higher blood glucose 60 min after glucose supplementation (**Figure 2A**). This suggests that muscle specific expression of SOD1^G93A^ determines a delay in glucose clearance. To substantiate this hypothesis, we analyzed the expression levels of Glut-4, a glucose receptor in muscle fibers, responsible for muscle glucose uptake. As shown in **Figure 2** protein levels of Glut4 was significantly down-modulated in the MLC/SOD1^G93A^ muscles (**Figure 2B** and Supplementary Figure S2), suggesting that the clearance delay of glucose could mainly be due to a decreased capacity of transgenic muscle to uptake glucose from circulation.

Glycolysis is regulated by several enzymes such as the Hexokinase 2 (HK2) and Phosphofructokinase 1/2, (PFK1, PFK2) that catalyze, respectively, the first and the second steps of glycolysis. In the asymptomatic animal model of ALS, the PFK activity is significantly down-modulated and muscle glucose metabolism is inhibited (Palamiuc et al., 2015).

To verify whether glycolysis was also altered in the MLC/SOD1^G93A^ mice, we analyzed the transcription levels of the Hexokinase 2 (HK2) and Phosphofructokinase 1/2, (PFK1, PFK2) in TA muscles of transgenic mice. As shown in **Figure 2**, differently from that observed in ALS mouse model (Shi et al., 2010), the glycolytic enzyme was significantly up-regulated in the muscle of MLC/SOD1^G93A^ mice compared to the Wt littermates (**Figures 2C–E**).

Pyruvate is the end product of Glycolysis and its levels are governed by the PDH that transforms Pyruvate into Acetyl-CoA. Acetyl-CoA enters the Krebs cycle and produces ATP and energy fuel for cells through oxidative phosphorylation. The Pyruvate kinase 4 (PDK4) is a negative regulator of PDH complex and inhibits PDH activity by phosphorylation (Denton et al., 1975). To investigate whether Pyruvate is efficiently converted into Acetyl-CoA we assessed the level of total and the phosphorylated isoform of PDH and the levels of PDK4. Interestingly we observed a significant accumulation of the phosphorylated isoform of PDH and a concomitant up-regulation of PDK4, indicating the inhibition of Acetyl-CoA synthesis (**Figures 2F,G** and Supplementary Figure S3). Moreover, as pyruvate accumulation stimulates the conversion of glucose to glycogen, we assessed the glycogen synthesis in the MLC/SOD1^G93A^ muscles. We observed a significant down-modulation of the inhibitory phosphorylated isoform of the Glycogen Synthase Kinase 3β (GSK3β) and a concomitant increase in intramuscular glycogen deposition, assayed by Periodic acid-Schiff (PAS) staining, in the TA muscle of MLC/SOD1^G93A^ mice compared to that of Wt littermates (**Figures 2H,I** and Supplementary Figure S4). Conversely, the mRNA level of Citrate Synthase (CS), the first enzyme of Krebs cycle, was comparable between Wt and MLC/SOD1^G93A^ muscles (**Figure 2J**), suggesting that alternative catabolic pathway, such as proteolysis or lipolysis, can guarantee sufficient Acetyl-CoA synthesis.

Overall these data suggest that muscle restricted expression of SOD1 mutant gene triggers the activation of the glycolysis process, which cannot efficiently contribute to oxidative phosphorylation and energy production.

### MLC/SOD1^G93A^ Transgene Is Associated With the Increase of the Lipid Handling Pathway

Various factors, including increased fatty acid (FA) use by β-oxidation, stimulate PDK4 expression (Jeong et al., 2012). The β-oxidation process is strictly regulated by the levels of its inhibitor MalonylCoA that is in turn induced by the Acetyl-CoA carboxylase (ACC). Acetyl-CoA carboxylase strongly controls lipid synthesis/degradation flux, and its activity is inhibited by phosphorylation.

**FIGURE 3:**
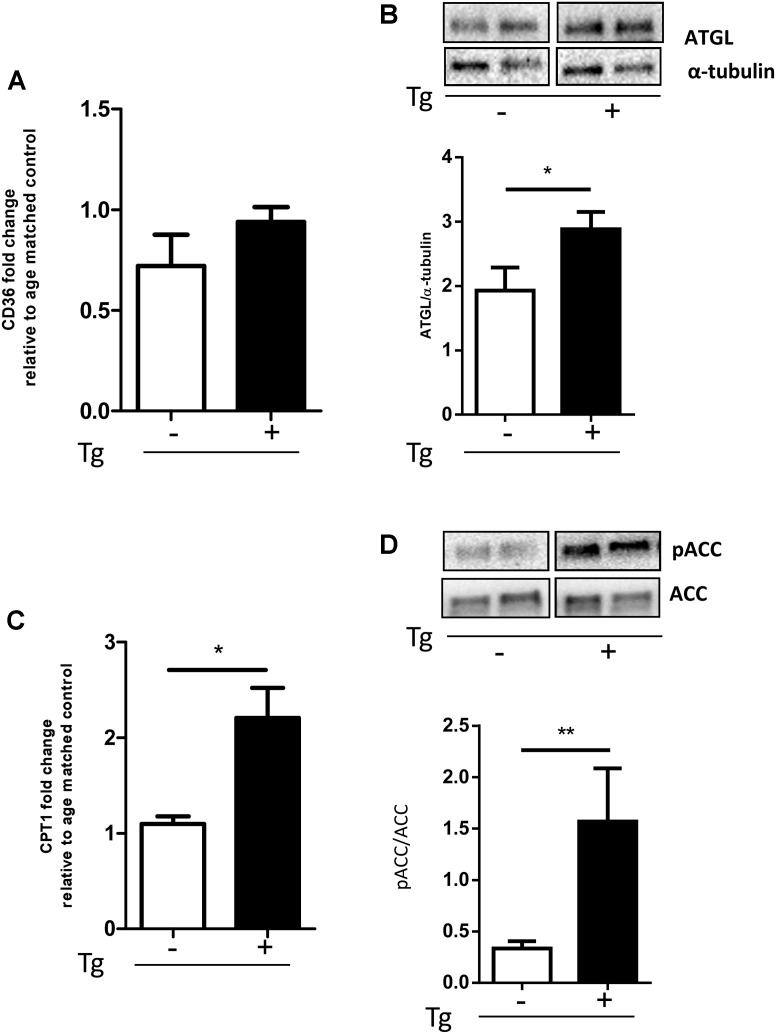
MLC/SOD1^G93A^ mice exhibit an increase of the lipid handling pathway. **(A)** Real-time PCR analysis of CD36 transcript in Wt and Tg mice (Wt, Tg *n* = 4,4); **(B)** Representative Western blot analysis of ATGL protein expression in both Wt and Tg mice. Lower panel shows densitometric analysis for ATGL expression relative to α-tubulin in Wt and Tg mice (^∗^*p* = 0.0336 Wt, Tg *n* = 9,12); **(C)** Real-time PCR analysis of CPT1 transcript in Wt and Tg mice (^∗^*p* = 0.0159 Wt, Tg *n* = 4,5.) **(D)** Representative Western blot analysis of pACC/ACC. Lower panel shows densitometric analysis for pACC expression relative to ACC in Wt and Tg mice (^∗^*p* = 0.0117 Wt, Tg *n* = 9,12). Data are represented as mean ± SEM. In **(B,D)** the lanes were run on the same gel but were noncontiguous.

To investigate muscle fibers oxidative metabolism, we studied the expression levels of key proteins of the FA regulation: CD36, a membrane translocase that promotes FA entry into the cells, ATGL, a lipase involved in lipid droplet degradation, and CPT-1B, responsible for the FA transfer into mitochondria. When compared to Wt littermates, the MLC/SOD1^G93A^ mice had a significant increase in the expression of all genes studied (**Figures 3A–C** and Supplementary Figure S5), including the pACC/ACC ratio (**Figure 3D** and Supplementary Figure S6), suggesting that lipid catabolism is stimulated and lipid synthesis is inhibited.

**FIGURE 4:**
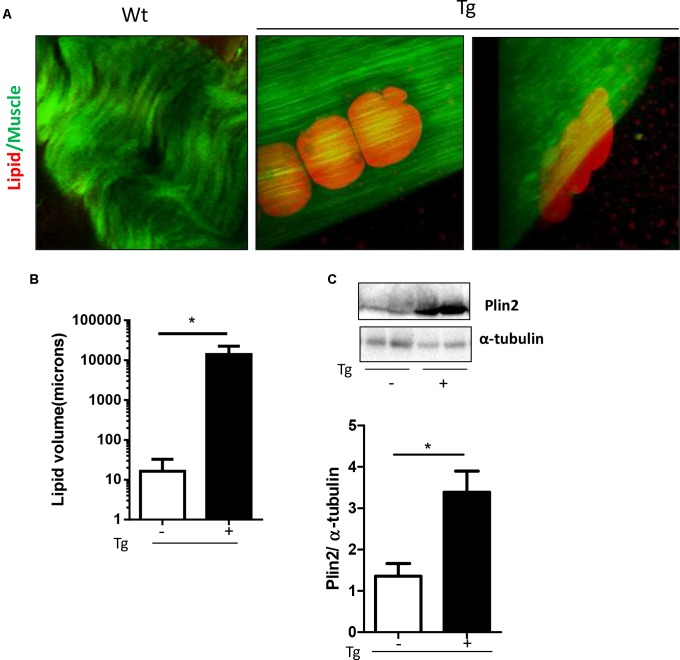
MLC/SOD1^G93A^ mice exhibit a significant accumulation of fat residues just outside fibers. **(A)** CARs microscopy analysis shows lipid droplets in EDL muscles of Tg mice compared to Wt littermates. **(B)** Analysis of total lipid volume outside fibers in EDL muscle of Wt and Tg mice (^∗^*p* = 0.0224 Wt, Tg *n*. microscope fields = 4,9). **(C)** Representative Western blot analysis of PLIN2 in both Wt and Tg mice. Lower panel shows densitometric analysis for PLIN2 expression relative to α-tubulin in Wt and Tg mice (^∗^*p* = 0.0159 Wt, Tg *n* = 5,5). Data are represented as mean ± SEM. In **(C)**, the lanes were run on the same gel but were noncontiguous.

Qualitative analysis on lipid droplet accumulation inside muscle fibers confirmed a preferential use of lipid of the transgenic muscle. As described in **Figure 4** no droplets were found inside muscle fibers; we observed a significant accumulation of fat residues just outside muscle fibers (**Figure 4A**). Quantitative analysis revealed a significant up-regulation of lipid droplet volume in the MLC/SOD1^G93A^ muscle compared to Wt littermates (**Figure 4B**). This data was further supported by western blot analysis for Perilipin 2 (PLIN2) expression, a coat protein of lipid droplet. As shown in **Figure 4C** we observed a significant up-regulation of Perilipin-2 in MLC/SOD1^G93A^ muscles compared to Wt ones (**Figure 4C** and Supplementary Figure S7).

These data suggest that muscle specific expression of mutant SOD1 induces a preferential recruitment of FAs to skeletal muscle tissue to sustain lipid flux into the skeletal muscle fibers and to guarantee FAs muscle availability and β-oxidation (Morales et al., 2017).

## Discussion

Our study demonstrates that perturbation in redox signaling cascades, induced by muscle specific expression of SOD1^G93A^, determines a muscle metabolic adaptation towards a more oxidative metabolism, demonstrating the activation of the slow-oxidative muscle fiber program and the preferential use of lipid fuel by the transgenic mice MLC/SOD1^G93A^.

Interestingly, we observed that muscle metabolic adaptation to oxidative stress does not involve significant changes in MyHC expression, in line with previous published works that have suggested a dissociation between metabolic and contractile adaptive response to different stimuli (Schiaffino and Reggiani, 2011). Indeed, it has been demonstrated that moderate endurance training can induce metabolic changes without concomitant changes in MyHC composition. Moreover, slow-type electrical stimulation in rat fast muscles can early abrupt MyHC-2B to MyHC-2X transcript switch (Kirschbaum et al., 1990), in response to external stimuli of muscle activity or inactivity (Ausoni et al., 1990).

Our data support the evidences that metabolic and contractile properties are controlled by distinct signaling pathways and provide insight into the mechanism controlling the regulation of fast-glycolytic and slow-oxidative gene program, induced by muscle specific expression of SOD1^G93A^. In particular, among the possible mechanisms that account for the modulation of different fast and slow muscle genes, our data support the involvement of miRNAs hosted in β/*slow* and *slow-tonic* Myosin locus.

Recent works have demonstrated that some muscle-specific microRNAs are located within Myosin genes and thus are co-expressed with these genes (van Rooij et al., 2009). Among these microRNAs, miR-499 is located in an intron of Myh7b and targets a transcriptional repressor of β/*slow* Myosin, namely Sox6, during muscle development (van Rooij et al., 2008). Nevertheless, it remains to elucidate whether muscle-specific miRNAs and Sox6, controls of fiber type diversification in developing muscle, is also involved in the maintenance of fiber type properties in adult skeletal muscle (van Rooij et al., 2009; Gan et al., 2013). Here we provide evidences that the oxidative insult induced by SOD1 mutant gene expression can determine the modulation of miR-499 regulative circuit toward a slow muscle fiber program, in muscle tissue of adult mice.

The shift of muscle fibers, observed in MLC/SOD1^G93A^ mice, toward the oxidative metabolism might represent a compensatory mechanism activated to cope the toxic effects of mutant SOD1 protein.

In the SOD1^G93A^ mice, which ubiquitously express the mutant gene (Gurney et al., 1994), the switch toward lipid use is considered an early sign of disease and occur early before symptomatic denervation (Palamiuc et al., 2015). It has been suggested that the early switch of glycolytic fibers toward oxidative phenotype is due to loss of connections of muscle fibers to large motoneurons, and to their subsequent reinnervation by slow motoneurons. Thus, these studies suggest that the oxidative switch of muscle fibers is a consequence of motor unit alteration, rather than an intrinsic property of muscle fibers plasticity that can change the metabolic properties independently from neuron physiology.

Here, we demonstrate that in the MLC/SOD1^G93A^ fibers, the metabolic oxidative switch occurs independently from motor neuron degeneration (Dobrowolny et al., 2018) and along with neuromuscular instability and deficiency in mitochondrial chain function (Dobrowolny et al., 2018). Considering the mitochondria defects and the oxidative damage induced by muscle specific SOD1 mutant gene expression (Dobrowolny et al., 2008, 2018), we can speculate that the oxidative change in energy fuel of MLC/SOD1^G93A^ fibers might reflect an adaptation to preserve muscle functionality (Blaauw et al., 2013). In addition, fiber type diversification may also reflect an adaptation to whole body metabolism and different pattern of activity. These observations are also in line of recent evidences that demonstrated how the up-regulation of Mir-499 and Mir-208b is associated with a reduced atrophy of type I fibers in muscle biopsies of ALS patients with slow progressive disease (Di Pietro et al., 2017).

The metabolic shift towards lipid use in the TA muscles in the ALS mouse model that ubiquitously express the SOD1 mutant gene (Gurney et al., 1994) is accompanied by lower levels of PFK activity in both presymptomatic and symptomatic stages of the disease (Palamiuc et al., 2015). These data are in apparent contrast with the up-regulation of both PFK1 and PFK2 observed in the MLC/SOD1^G93A^ mice. In the ALS mouse model at the presymptomatic stage of the disease, higher levels of Pyruvates correlate with a decrease of PFK activity; conversely at the end stage of the disease low levels of Pyruvate correlates with low levels of PFK activity, due to the inhibitory feed-back of Pyruvate. Therefore, although the glycolysis is enhanced in the MLC/SOD1^G93A^ mice, Pyruvate levels could be still not sufficient to induce a Pyruvate dependent inhibitory signal of glucose catabolism. Moreover, Pyruvate can be used in other compensative pathways that do not involve mitochondria machinery, such as glycogen synthesis or anaerobic glycolysis.

In conclusion, our data reveal that localized expression of SOD1^G93A^ induces metabolic changes in skeletal muscle independently of motor neuron degeneration and further indicate that skeletal muscle is likely an important target for therapeutic intervention in ALS.

## Ethics Statement

The experimental protocol was approved by the Ethical Committee (OPBA) of the Sapienza University of Rome and by the Italian Ministry of Health, Italy (26/2014, authorization number 609/2015-PR).

## Author Contributions

AM conceptualized the study, contributed the resources, supervised the project, and did the project administration. GD, EL, MM, LB, AN, and BMS contributed to the methodology. GD, EL, MM, BMS, and AM contributed to the validation. GD, EL, MM, LB, BMS, and AM carried out the formal analysis and curated the data. GD, EL, LB, MM, and BMS contributed to the investigation. AM and GD wrote the original draft, the review, and edited the manuscript. GD, EL, LB, MM, BMS, and AM contributed to the observation. AM and BMS acquired the funding.

## Conflict of Interest Statement

The authors declare that the research was conducted in the absence of any commercial or financial relationships that could be construed as a potential conflict of interest.
