# Supplementary material for: Metabolic Changes Associated With Muscle Expression of SOD1G93A
Source: Front Physiol. 2018 Jul 10;9:831. doi: 10.3389/fphys.2018.00831 (PMC6048270; doi:10.3389/fphys.2018.00831)
Supplement: Supplementary file 1 [file Data_Sheet_1.pdf]

Supplementary Material

Metabolic changes associated with muscle expression of $SOD1^{G93A}$

Gabriella Dobrowolny[1,2,#,*], Elisa Lepore[1,2,#], Martina Martini[1], Laura Barberi[1], Abigail Nunn[2], Bianca Maria Scicchitano[3], Antonio Musarò[1,2,*]

*** Correspondence:** Correspondence: Antonio Musarò or Gabriella Dobrowolny, DAHFMO-Unit of Histology and Medical Embryology, Sapienza University of Rome, Via Scarpa 16 Rome 00161.

E-mail: antonio.musaro@uniroma1.it; gabriella.dobrowolny@uniroma1.it

1 Supplementary Figures and Tables

1.1 Supplementary Figures

Supplementary Figure 1.

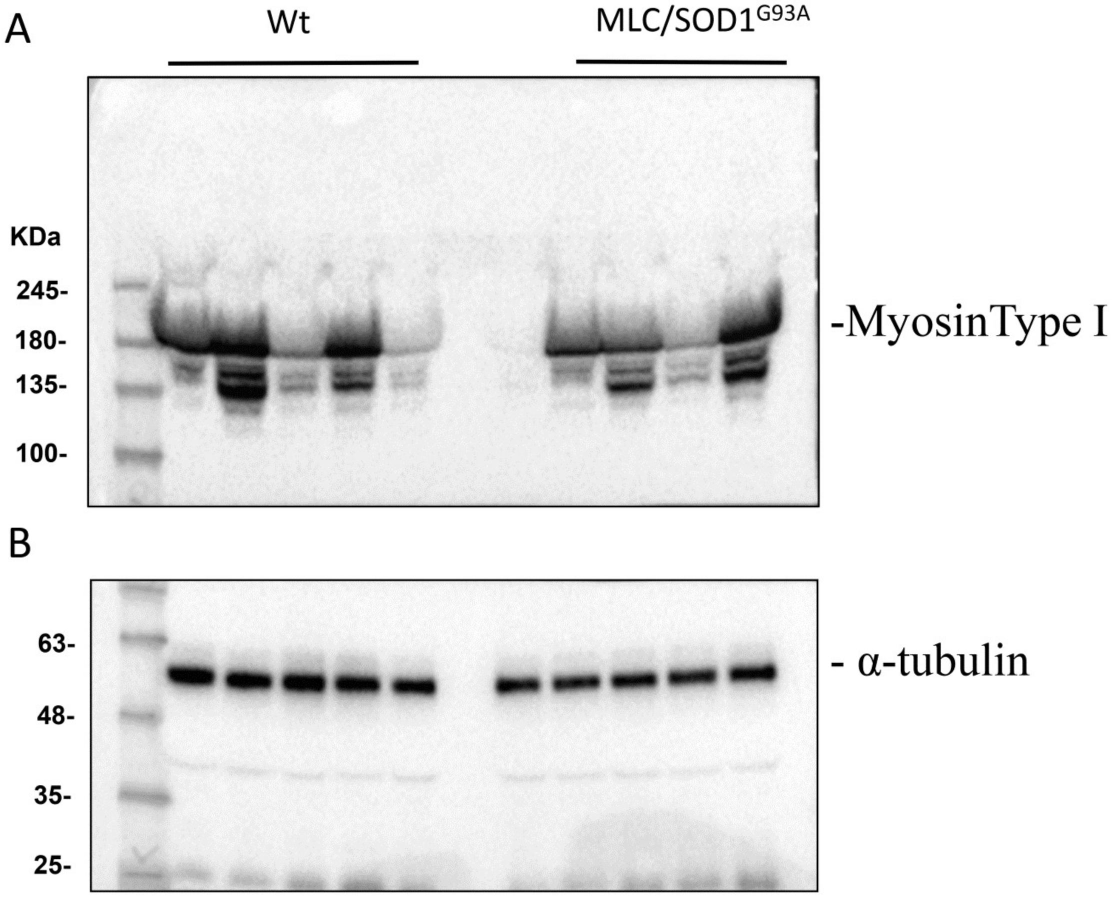

SUPPLEMENTARY FIGURE 1. Western blot analysis of (A) Myosin Type I and (B) α-tubulin expression in wild type (Wt) and MLC/SOD1^{G93A} transgenic mice; α-tubulin is used as a control for protein loading. Correspondent cropped bands and densitometric analysis are show in Figure 1A.

Supplementary Figure 2.

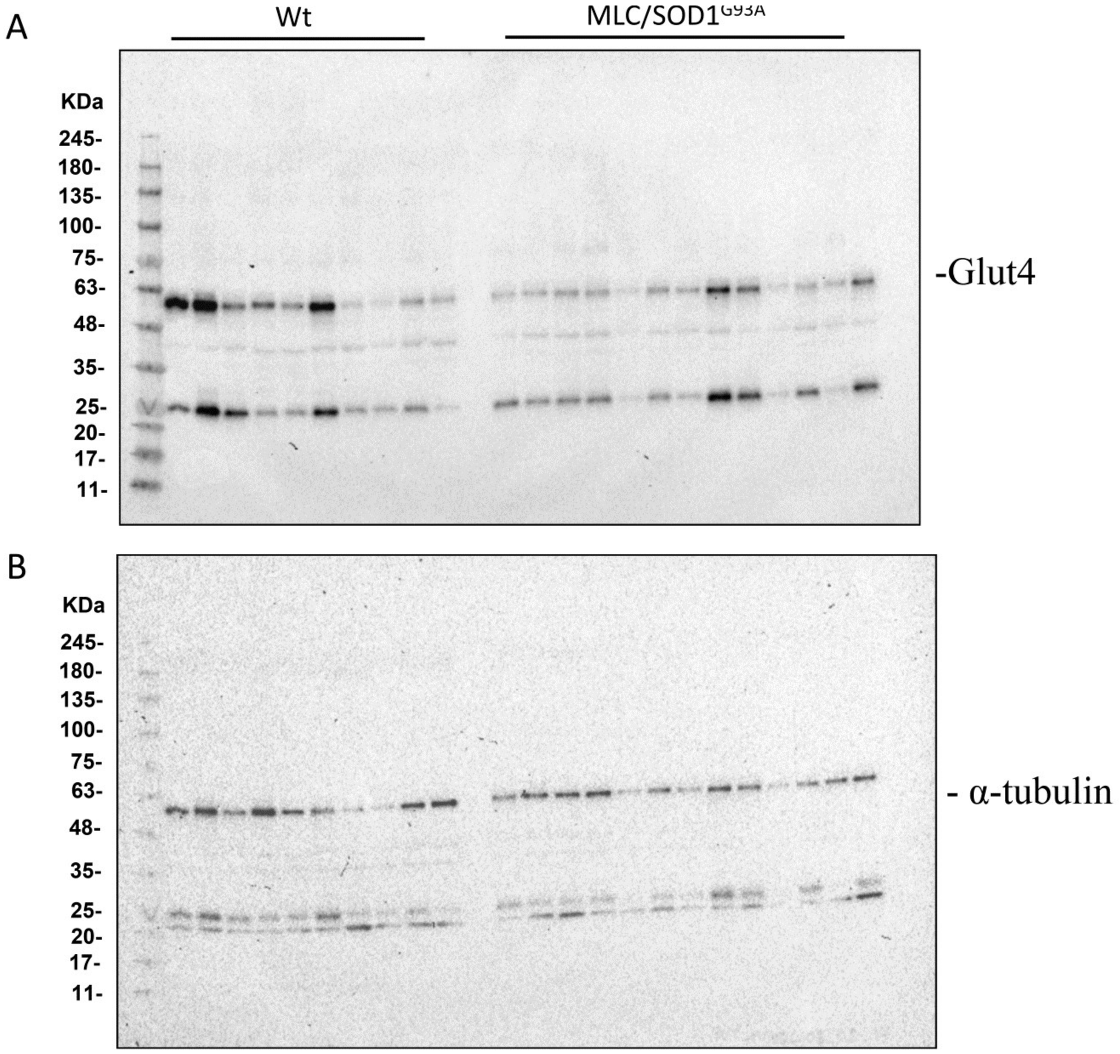

SUPPLEMENTARY FIGURE 2. Western blot analysis of (A) GLUT4 and (B) α-tubulin expression in wild type (Wt) and MLC/SOD1^{G93A} transgenic mice; α-tubulin is used as a control for protein loading. Correspondent cropped bands and densitometric analysis are show in Figure 2B.

Supplementary Figure 3.

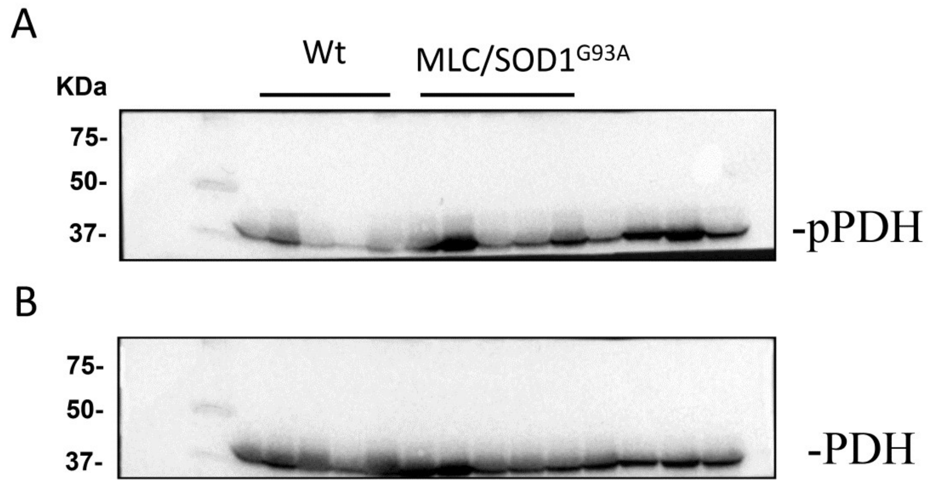

SUPPLEMENTARY FIGURE 3. Western blot analysis of (A) pPDH and (B) PDH expression in wild type (Wt) and MLC/SOD1^{G93A} transgenic mice; Correspondent cropped bands and densitometric analysis are show in Figure 2G.

Supplementary Figure 4

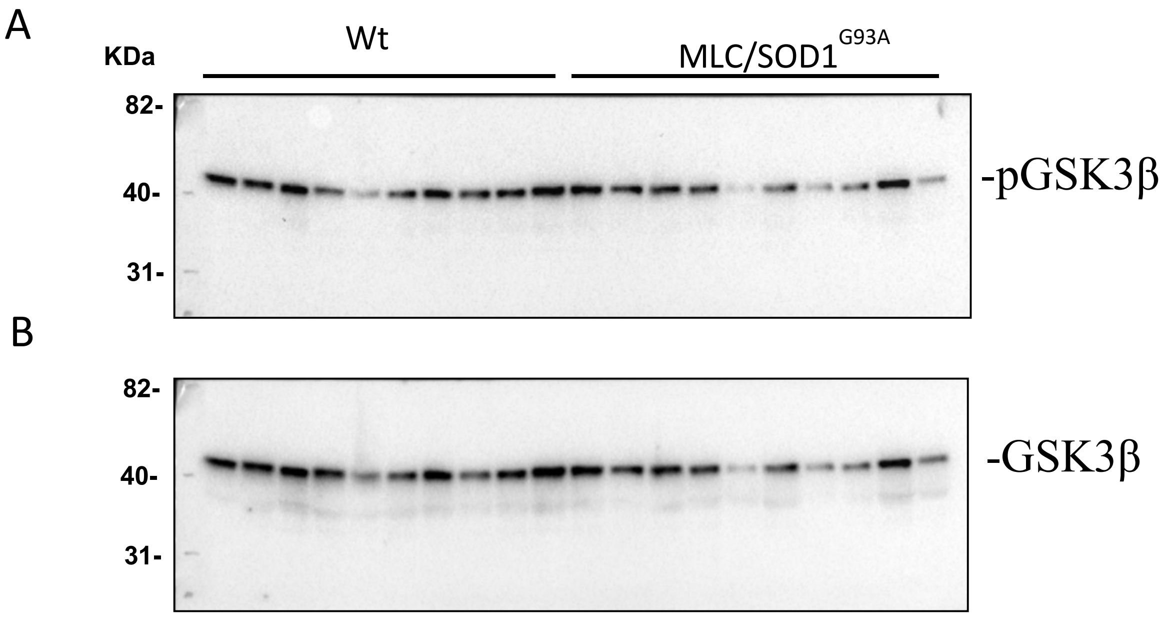

SUPPLEMENTARY FIGURE 4. Western blot analysis of (A) pGSK3β and (B) GSK3β expression in wild type (Wt) and MLC/SOD1^{G93A} transgenic mice; Correspondent cropped bands and densitometric analysis are show in Figure 2H

Supplementary Figure 5.

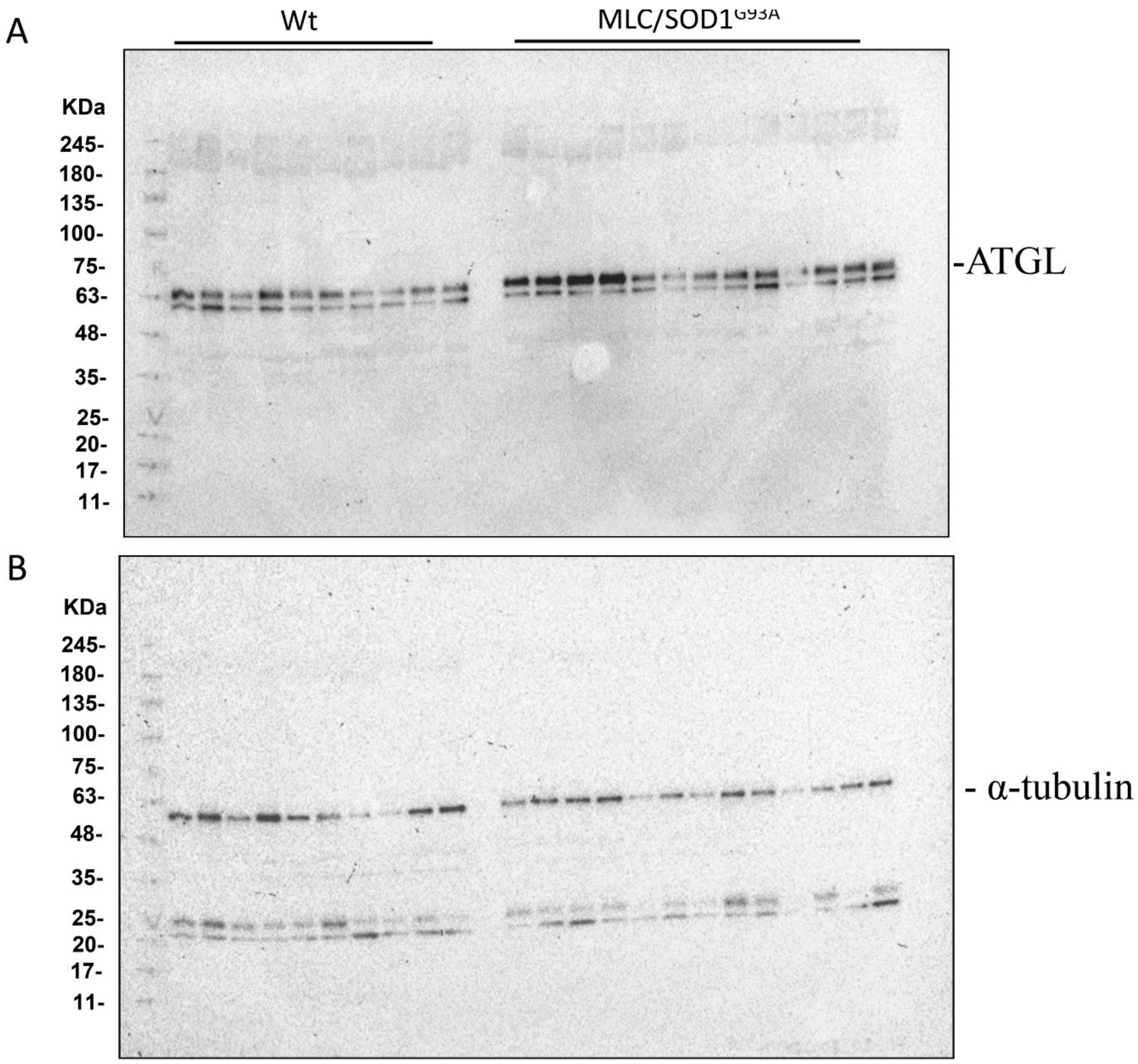

SUPPLEMENTARY FIGURE 5. Western blot analysis of (A) ATGL and (B) α-tubulin expression in wild type (Wt) and MLC/SOD1^{G93A} transgenic mice; α-tubulin is used as a control for protein loading. Correspondent cropped bands and densitometric analysis are show in Figure 3B.

Supplementary Figure 6.

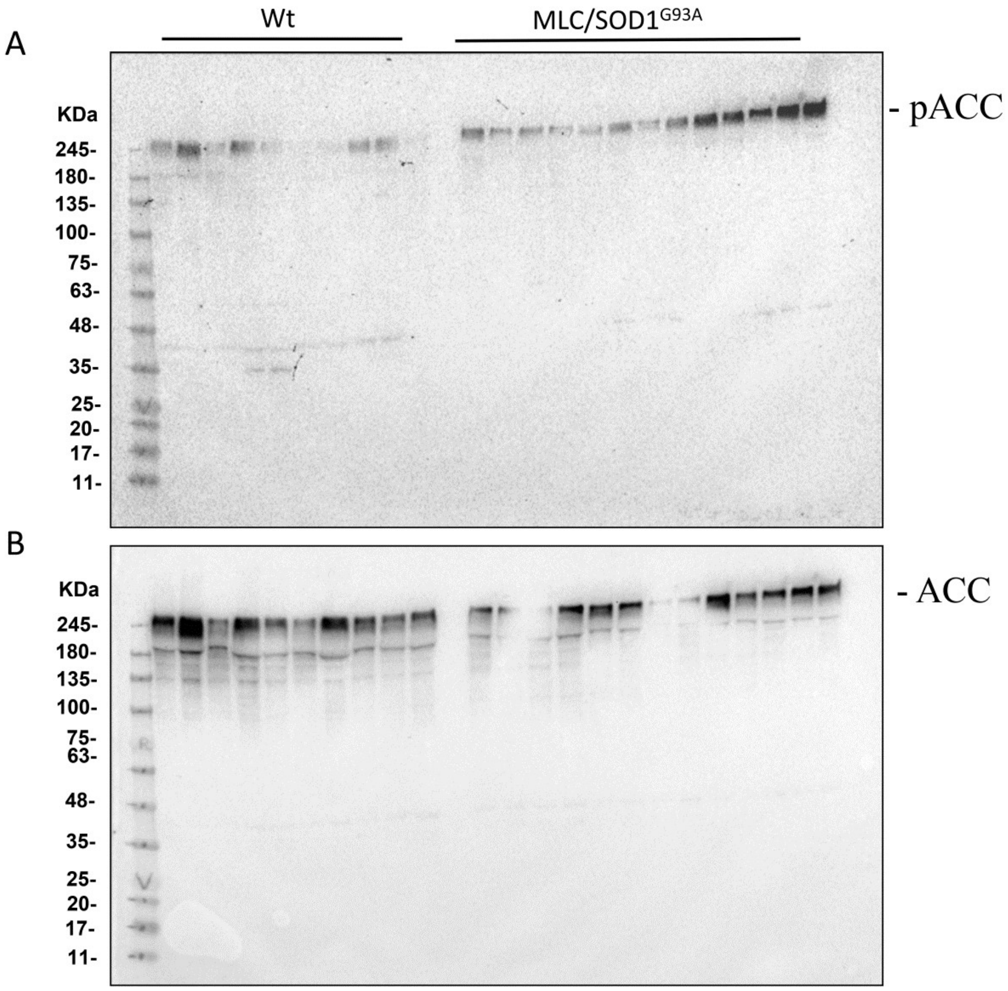

SUPPLEMENTARY FIGURE 6. Western blot analysis of (A) pACC and (B) ACC expression in wild type (Wt) and MLC/SOD1^{G93A} transgenic mice. Correspondent cropped bands and densitometric analysis are show in Figure 3D.

Supplementary Figure 7.

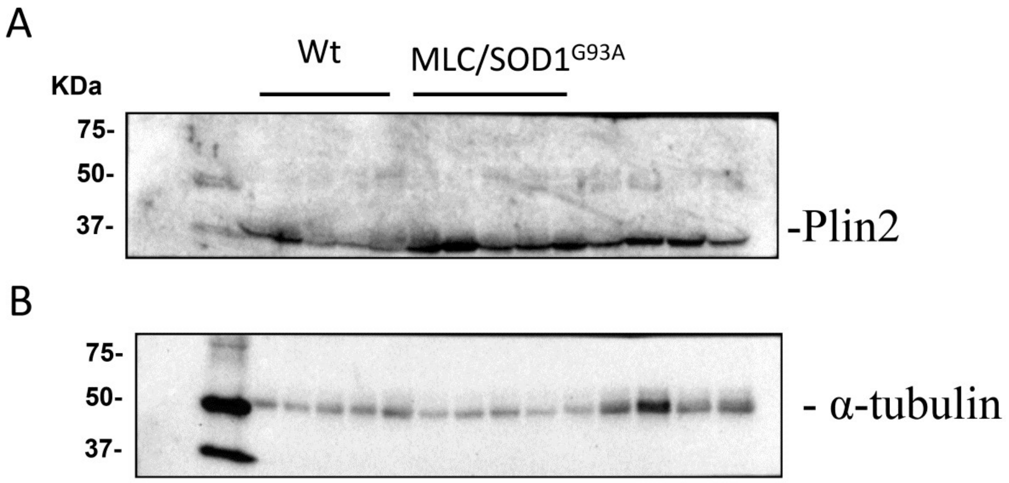

SUPPLEMENTARY FIGURE 7. Western blot analysis of (A) Plin2 and (B) α-tubulin expression in wild type (Wt) and MLC/SOD1^{G93A} transgenic mice; α-tubulin is used as a control for protein loading Correspondent cropped bands and densitometric analysis are show in Figure 4C.
